# Epithelial–Mesenchymal Transition and Stress Adaptations Underlie Yttrium-90 Resistance in Liver Cancer Cell Lines

**DOI:** 10.1158/2767-9764.CRC-25-0627

**Published:** 2026-01-22

**Authors:** Alexander Zheleznyak, Dhanusha Duraiyan, Prasanth Thunuguntla, Jessica Camacho, Shuang Wu, Jingxia Liu, Jason D. Weber, Daniel L.J. Thorek, Benjamin S. Strnad, Ryan C. Fields, Matthew A. Ciorba, Christine Yoon, Valerie Blanc, Nicholas Davidson, Jessica Silva-Fisher, Christopher D. Malone

**Affiliations:** 1 Mallinckrodt Institute of Radiology, Washington University School of Medicine, St. Louis, Missouri.; 2 Division of Oncology, Department of Medicine, Washington University School of Medicine, St. Louis, Missouri.; 3 Department of Surgery, Washington University School of Medicine, St. Louis, Missouri.; 4 Siteman Cancer Center, Washington University in St. Louis, St. Louis, Missouri.; 5 Division of Gastroenterology, Department of Medicine, Washington University School of Medicine, St. Louis, Missouri.; 6 Albert Einstein School of Medicine, Bronx, New York.

## Abstract

Molecular signatures predict prognosis in hepatocellular carcinoma, but their relevance to transarterial radioembolization (TARE) with yttrium-90 (^90^Y) is unknown. We aimed to identify baseline and treatment-induced pathways associated with response and nominate biomarkers. Ten transcriptomically diverse human liver cancer cell lines were exposed to escalating activities of glass ^90^Y microspheres for 10 days. Normalized AUC values quantified sensitivity. Whole-transcriptome RNA sequencing at baseline and after treatment was analyzed with elastic net regression and gene set enrichment. Findings were corroborated by qRT-qPCR and exploratory analysis of pretreatment tumor samples from patients undergoing TARE. Liver cancer cell line responses to ^90^Y were heterogeneous, with resistance aligning to Hoshida S1 and cholangiocarcinoma-like subtypes. Epithelial–mesenchymal transition (EMT) and adhesion pathways were enriched in resistant lines, with *CD44* and *ITGA3/*α3β1 emerging as candidate markers, corroborated by RNA and protein expression. After ^90^Y exposure, resistant lines upregulated IFNγ/α, TNFα/inflammatory, and antigen presentation–related pathways, whereas sensitive lines downregulated these pathways along with DNA repair and oxidative phosphorylation. In an exploratory patient cohort, higher tumor *CD44* expression trended with early progression. In conclusion, liver cancer cell lines display marked biological heterogeneity in response to ^90^Y. Baseline EMT/adhesion signatures and stress response pathways nominate *CD44* and *ITGA3*/α3β1 as candidate biomarkers of resistance. These findings delineate molecular programs of β-emitter radioresistance and identify candidate pathways for future targeting.

**Significance:**

TARE with ^90^Y is widely used for liver cancer, yet its molecular determinants of response are poorly understood. Using a diverse panel of liver cancer cell lines, we identify EMT, adhesion, and stress response pathways associated with resistance. These findings highlight candidate biomarkers and molecular vulnerabilities that may guide future therapeutic strategies and patient selection.

## Introduction

Hepatocellular carcinoma (HCC) is a rapidly increasing cause of cancer-related death worldwide, with a 5-year survival of less than 20% (1). Among effective locoregional therapies, transarterial radioembolization (TARE) involves the transarterial delivery of microspheres embedded with the pure β-emitting tumoricidal radioisotope yttrium-90 (^90^Y) to liver tumors (2). With growing evidence of efficacy, it has seen an increasing role in managing HCC with incorporation into global practice guidelines (3–9).

TARE is highly effective in well-selected patients, producing durable responses with minimal toxicities. However, outcomes can be heterogeneous in patients with more aggressive disease who remain at risk for early out-of-field progression (OFP). Global multicenter data have demonstrated a positive relationship between tumor-absorbed dose (TAD) and treatment response, but nonresponse is still seen in a subset of patients even when guideline-concordant thresholds are achieved (10). These discrepancies support the hypothesis that the underlying biological heterogeneity of HCC significantly influences treatment outcomes. Furthermore, the concept that residual tumor cells persist after treatment and ultimately drive treatment-refractory recurrence underscores the importance of identifying molecular markers and pathways that predispose to treatment resistance (11, 12). Such insights may ultimately enable rational design of strategies to improve tumor eradication.

Several large-scale transcriptomic analyses have defined prognostic molecular subclasses of HCC, including Hoshida and the NCI, among others (13–16). However, their relationship and applicability to specific therapies such as TARE remain largely unexplored. In addition, this molecular granularity is not captured by contemporary staging and treatment guidelines, which are limited by anatomic factors, including tumor size and number, presence of vascular invasion or metastases, or clinical indicators, such as performance status and liver function (3). The distinctive physical characteristics of ^90^Y, including the continuous β-particle emission over its decay (Emax 2.28 MeV, mean 0.93 MeV, and 64.2-hour half-life), impart unique radiobiological properties, but their associated molecular signatures have not been explored (17–20). There remains a paucity of fundamental studies investigating biological determinants of tumor sensitivity or resistance to β-emitting radiopharmaceuticals and even less insights into tumor responses across genetically diverse HCC models. To our knowledge, there is a lack of work profiling molecular correlates of β-emitter resistance in liver cancer.

In this study, we employed a panel of 10 transcriptomically diverse human liver cancer cell lines to characterize biological determinants of response to ^90^Y-microsphere exposure. Integrating functional dose–response assays with transcriptomic profiling, we sought to identify baseline and treatment-induced pathways associated with ^90^Y resistance. These findings aim to generate hypotheses about molecular mechanisms of ^90^Y response that may guide future biomarker validation and translational investigation in patients undergoing TARE.

## Materials and Methods

### Cell lines and reagents

**Figure 1.**
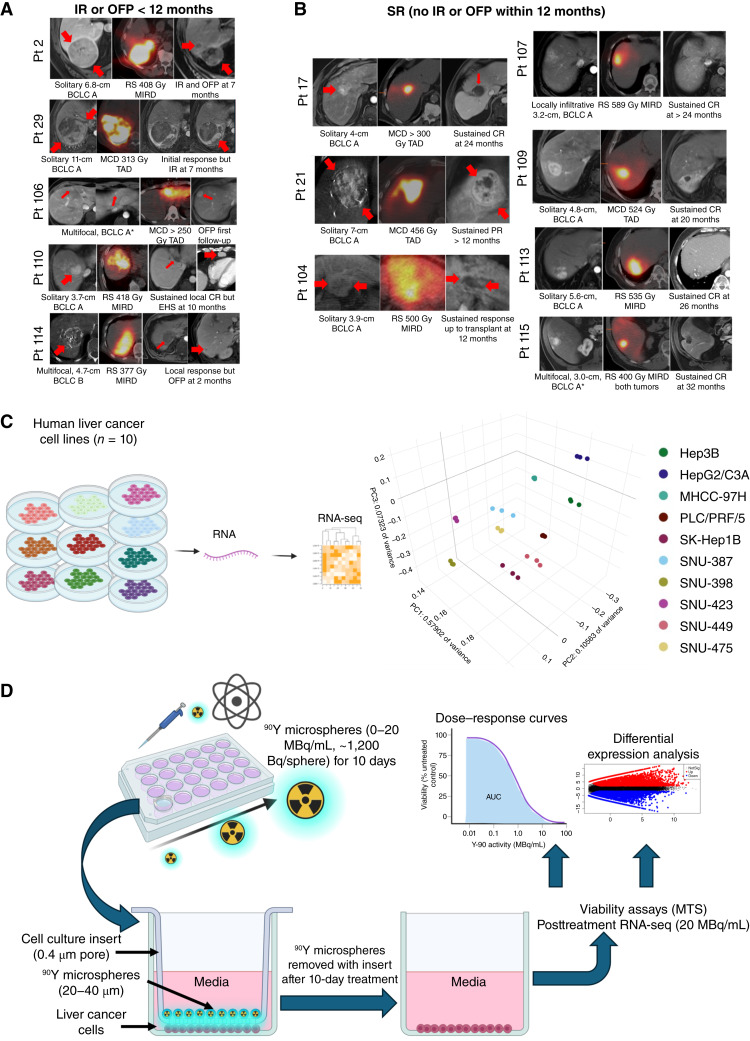
Study rationale, design, and experimental workflow. Heterogeneity of response in a preliminary cohort of patients with HCC showing (**A**) cases of IR or OFP within 12 months of ^90^Y-RE vs. (**B**) sustained responders (SR), defined as no IR or OFP within 12 months of treatment. Each row indicates one patient, with panels from left to right demonstrating baseline contrast-enhanced MR (red arrows highlighting tumors of interest), immediate post-therapy ^90^Y SPECT/CT and treatment details, including reported dose, and follow-up MR at indicated time point showing outcomes. **C,** A panel of 10 human liver cell lines spanning the transcriptomic spectrum as demonstrated by PCA of baseline RNA expression profiles. **D,** Cells were treated at escalating activities of ^90^Y microspheres (0–20 MBq/mL) for 10 days in 24-well cell culture plates using 0.4-μm pore inserts to physically separate microspheres to enable removal for downstream assays. Cell viability (MTS) was measured in triplicate per condition across activity range. Baseline and posttreatment (20 MBq/mL) RNA-seq followed by differential expression analysis was performed in biological triplicates in selected (7) cell lines presenting a spectrum of sensitivity to ^90^Y microsphere treatment. * for multifocal BCLC A, only 1 tumor shown for illustrative purposes. BCLC, Barcelona Clinic Liver Cancer; EHS, extrahepatic spread; MCD, multicompartment dosimetry; MIRD, medical internal radiation dose; Pt, patient; RS, radiation segmentectomy.

An overview of the experimental design and setup is demonstrated in Fig. 1. The following human liver cancer cell lines were obtained from the ATCC: SK-Hep1 (RRID: CVCL_0525), Hep-3B2 (RRID: CVCL_0326), HepG2/C3A (RRID: CVCL_1098), PLC/PRF/5 (RRID: CVCL_0485), SNU-387 (RRID: CVCL_0250), SNU-423 (RRID: CVCL_0366), SNU-449 (RRID: CVCL_0454), and SNU-475 (RRID: CVCL_0497). SNU-398 (RRID: CVCL_0077) was a generous gift from Terence Gade, MD, PhD (acquired from ATCC, Penn Image Guided Interventions Lab, University of Pennsylvania). MHCC-97H (RRID: CVCL_4972) was a generous gift from Ichor Life Sciences. This panel of 10 human liver cancer cell lines was selected given its representation across transcriptomic subtypes and varying degrees of malignant potential as described by Caruso and colleagues (21). Although SK-Hep-1 and HepG2 are derived from adenocarcinoma and hepatoblastoma, respectively, both are liver derived and frequently used in liver cancer research. Their inclusion provides biological diversity relevant to tumors with mesenchymal, cholangiocarcinoma-like (C1), or developmental traits (22, 23). To ensure the cells were free of contamination, *Mycoplasma* testing was performed by the Washington University Genome Engineering and Induced Pluripotent Stem Cell Core using the MycoAlert PLUS and MycoStrip kits. Cells were directly obtained from ATCC or Ichor Life Sciences in 2022 and the latest testing was conducted in 2022. Cell line identity was authenticated by ATCC using short tandem repeat profiling prior to distribution. Cells were expanded and used for experiments within five to six passages after thawing the original vials. Details and overview of the cell lines and culturing conditions used in this study are in Supplementary Methods and Supplementary Table S1.

### ^90^Y microspheres

^90^Y-impregnated glass microspheres (TheraSphere) were obtained from Boston Scientific through a material transfer agreement and were manufactured according to proprietary methods (24). All experiments were consistently performed 5 days after calibration to ensure uniform specific activity per microsphere (∼1,200 Bq/sphere).

### Cell viability assay

The approach for assessing cell viability after ^90^Y microsphere treatment was adapted from established methods used for radionuclide-based treatments (25–27). Additional details can be found in Supplementary Methods. Standing inserts were used to enable removal of ^90^Y microsphere after 10-day treatment for downstream assays. This time point was selected because it was more than three half-lives of ^90^Y decay (64.2 hours) and well exceeded the published doubling times. ^90^Y microspheres were added to the inserts at activity concentrations of 0 to 20 MBq/mL. Cell viability was determined using the CellTiter 96 AQueous One Assay (Promega, RRID: SCR_006724). The integral area under the curve (AUC_Δ_) was calculated using trapezoidal approximations, with differences assessed with the Wilcoxon rank, and the AUC_Δ_ of each cell line to AUC representing 100% survival/resistance control (AUC_R_) was calculated to obtain normalized AUC (nAUC) according to nAUC = AUC_Δ_/AUC_R_ (25–27)_._ The nAUC represents an integrated measure of treatment response across the range of ^90^Y activities, providing a quantitative summary of overall viability rather than relying on a single dose-point estimate.

### RNA sequencing and analysis

Total RNA sequencing (RNA-seq) was conducted for differential expression analysis after ^90^Y treatment on select cell lines (*n* = 7, representing a range of sensitivity to ^90^Y based on cell viability experiments) after treatment with 0 and 20 MBq/mL ^90^Y microspheres as described above. After 10 days of treatment, the cells were mechanically dissociated from the wells and submitted to the Tissue Procurement Core at Washington University School of Medicine (RRID: SCR_004876) for RNA extraction. RNA from each cell line was isolated according to standard methods. This was also performed for all 10 untreated cell lines for baseline transcriptomic analysis. Detailed RNA-seq and differential expression pipelines are available in the Supplementary Methods section. Experiments and sequencing were done in biological triplicates independent of the cell viability assays.

The correlation of baseline transcriptomic profile with previously established HCC signatures, including Hoshida, cholangiocarcinoma signature (CC), and hepatoblastoma HB-16 was performed for each cell line using nearest template prediction (14, 22, 23). To enable compatibility with published gene expression signatures, raw RNA-seq data were transformed to microarray-like distributions using the voom function in the limma package (RRID: SCR_010943), which applies log-count transformation and precision weighting. These signatures were selected because of their use in concurrent HCC genomic cohort studies (Hoshida) and association with cancer stemness (CC and HB-16).

### Western immunoblot assay

Western immunoblot assay was used to determine baseline expression of CD44 and α3β1 integrin in PLC/PRF/5, SNU-398, and SK-Hep1 untreated cells. See Supplementary Methods for further details.

### Elastic net regression analysis

Associations between gene mRNA expression levels and the ^90^Y microsphere sensitivity profiles across the 10 HCC lines were identified using elastic net regression (EN) combined with a bootstrapping procedure. The score for each gene was computed based on the frequency of positive and negative coefficients. Genes with a score of ≥0.7 were retained and considered predictive of the ^90^Y-resistant phenotype, resulting in 58 genes. Further details can be found in Supplementary Methods.

### qRT-PCR validation

Validation of gene expression changes representing major biological pathways after ^90^Y treatment, as informed by our gene set enrichment analysis (GSEA) analysis, was performed using qRT-PCR in independent experiments. SK-Hep1, SNU-398, and PLC/PRF/5 cells were treated with 0 and 20 MBq/mL ^90^Y microspheres as described above. Technical triplicates were performed for each condition, and biological replicates were performed when feasible. Primer sequences are available in Supplementary Table S2. qRT-PCR output was normalized to a housekeeping gene (*RPL32*).

### Patient cohort

A small, exploratory cohort 12 patients with HCC who had undergone TARE with available pretreatment tumor between 2021 and 2024 was identified for directional validation of *CD44* expression. This was conducted in accordance with both Declarations of Helsinki and Istanbul after Institutional Review Board (IRB) approval at our institution, and written informed consent was obtained. Available pretreatment tissue was obtained from either formalin-fixed, paraffin-embedded blocks archived from prior biopsies or prospectively obtained as frozen tissue in patients undergoing biopsy for suspected HCC. Background liver tissue was also obtained when available. RNA extraction and quantification were performed using standardized methods and are detailed in Supplementary Methods and primer sequences are detailed in Supplementary Table S2. *CD44* gene expression was normalized to that of the corresponding background liver. If a tumor specimen did not have a matched normal liver pair, it was normalized to the average of normal liver values across our cohort.

Early- or intermediate-stage patients were treated with ^90^Y-RE with glass microspheres (TheraSphere, Boston Scientific) using contemporary dosimetry guidelines, consisting of >400 Gy to perfused volume for ablative radiation segmentectomy intent or TAD >205 Gy with multicompartment dosimetry in cases of larger tumor burden (5, 6). Clinical and demographic data were recorded and summarized in Supplementary Table S3. Patients were classified as achieving sustained response (SR) if they were free from in-field recurrence (IR) or OFP for at least 12 months after initial treatment. No patients received systemic therapy, including immune checkpoint inhibitors, either prior to TARE or during the follow-up period. OFP within 1 year was used as an endpoint for surrogate biology of the primary tumor given the hypothesis that Hoshida S1 tumors were associated with early recurrence after surgical resection (14). Normalized tumor CD44 expression was compared between the SR and IR/OFP cohorts. These analyses were considered exploratory given the limited sample size and were intended to illustrate the potential translational relevance of *in vitro* findings rather than to establish statistical validation.

### Data analysis and statistics

Experiments were repeated three times (biological replicates), with each condition performed in triplicate to control for the intraexperimental variability. The experiments were not randomized, and the analysis was not blind. Genomic studies were conducted using triplicate samples for each condition. Data visualization and statistical analyses were performed using GraphPad Prism 10.4 (GraphPad Software, Inc., RRID: SCR_002798) and Microsoft Excel (RRID: SCR_016137). For data with two groups and one variable, a two-tailed unpaired *t* test was used. Data with one variable and multiple groups were analyzed using a one-way ANOVA, followed by Tukey or Dunnett multiple comparisons test to determine the adjusted *P* value. The correlation of cell line transcriptomic signature (i.e., Hoshida subclass) and nAUC was performed using the Kruskal–Wallis rank-sum test. Data with two variables and multiple groups were analyzed with a two-way ANOVA and Tukey multiple comparisons test to determine the adjusted *P* value. The data were expressed as mean ± SEM when representing more than one independent experiment unless indicated otherwise. Differences at the 95% confidence level (*P* < 0.05) were statistically significant.

### Statement of ethics

This was conducted through IRB approval (IRB ID# 201111078) at our institution, and informed consent was obtained for retrieval of tumor or liver tissue.

## Results

### Study design and experimental setup

HCC exhibits marked genetic heterogeneity, which likely contributes to variable response to β-emitting radionuclide therapy. We illustrate this heterogeneity in a preliminary cohort of patients with HCC treated with TARE, in which divergent responses were observed despite meeting guideline-concordant dosimetry thresholds (Fig. 1A and B). To model this heterogeneity systematically, we screened a panel of liver cancer cell lines spanning diverse transcriptomic backgrounds as demonstrated by principal component analysis (PCA) of baseline RNA expression (Fig. 1C). To understand the relationship between β-emitting radionuclide therapy and the transcriptomic signature, we designed a workflow to treat selected liver cancer cell lines with ^90^Y microspheres contained in the specialized “standing” well inserts (Fig. 1D). The distance between the bottom of the insert and the cells was 1 to 2 mm. The inserts allowed media exchange through the porous membrane with 0.4-μm pores while retaining the microspheres without significant attenuation of the emitted β particle and recapitulated the average distance achieved during TARE. Additionally, this design allowed immediate processing of the cells after treatment and removal of the inserts as residual radioactivity and microspheres significantly affect downstream assays utilizing luminescence or absorbance readouts.

### Liver cancer cell lines show variable sensitivity to ^90^Y treatment

**Figure 2.**
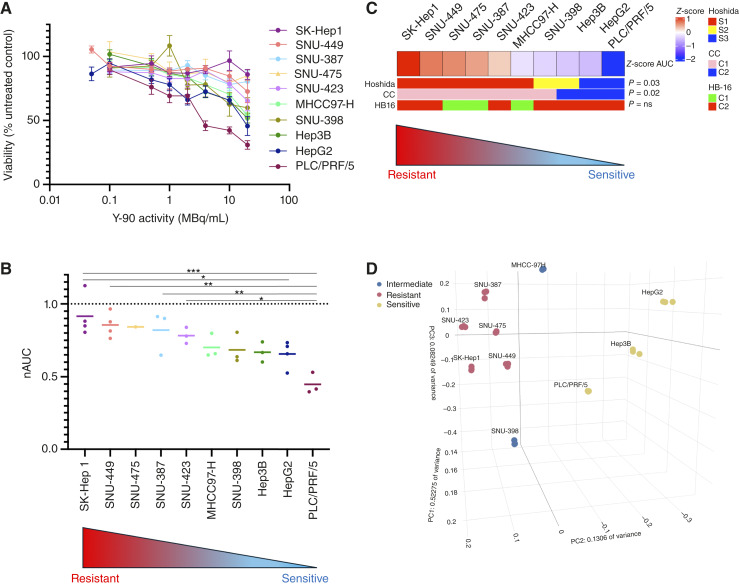
Heterogeneity of response to ^90^Y microsphere treatment across human liver cancer cell lines. **A,** Dose–response curves of cell viability after 10-day treatment to escalating ^90^Y microsphere activities (0–20 MBq/mL) in each of 10 cell lines. Each point represents the mean surviving fraction relative to untreated baseline control across all independent experiments (error bars: SEM). **B,** For each experiment, the area under the dose–response curve was calculated and normalized to yield nAUC (0 = sensitive and 1 = resistant). Cell lines are ordered left to right by decreasing nAUC (increased sensitivity). Horizontal bar indicates mean nAUC for each cell line across experiments. Group differences were assessed by one-way ANOVA with a Tukey multiple comparisons test (*, *P* < 0.05; **, *P* < 0.01; ***, *P* < 0.001). **C,** Relationship between response to ^90^Y and established HCC transcriptomic subtypes. Cell lines were assigned to select HCC transcriptomic subtypes by nearest template prediction. nAUC distributions differed by subtype, with Hoshida S1 and C1 (cholangiocarcinoma-like) subtypes associated with ^90^Y resistance (*P* < 0.05, Kruskal–Wallis rank-sum test). No correlation with the hepatoblastoma HB-16 signature was observed. **D,** PCA of RNA baseline expression profiles of all cell lines demonstrates clustering of the five most resistant cell lines by nAUC (red: SK-Hep1, SNU-449, SNU-475, SNU-387, and SNU-423) along PC2/PC3 (13.1%/8.2% variance), with clear separation of the three most ^90^Y-sensitive cell lines (yellow: PLC/PRF/5, Hep3B, and HepG2) along PC2.

Each cell line was exposed to escalating activities of ^90^Y microspheres (0, 0.1, 0.5, 1, 2, 4, 10, and 20 MBq/mL), corresponding to absorbed doses of 0 to 1,000 Gy via the medical internal radiation dose formalism and consistent with clinical ranges (10). The viability of each cell line was assessed by measuring the metabolic activity of the surviving cell fraction after 10 days of treatment. As shown in Fig. 2A, cell lines exhibited heterogeneous sensitivity to ^90^Y compared with the untreated control. nAUC values differed significantly across cell lines (one-way ANOVA, *P* = 0.0005), indicating variable resistance phenotypes (Fig. 2B). The most resistant lines, SK-Hep1 (nAUC = 0.915 ± 0.14), SNU-387 (nAUC = 0.733 ± 0.15), SNU-475 (nAUC = 0.842 ± 0.04), SNU-449 (nAUC = 0.877 ± 0.09), and SNU-423 (nAUC = 0.782 ± 0.06), corresponded with S1 Hoshida and C1 transcriptomic subclasses but not with the hepatoblastoma HB-16 (22, 23, 28). On the other hand, the most sensitive lines, HepG2 (nAUC = 0.655 ± 0.09), Hep3B (nAUC = 0.669 ± 0.07), and PLC/PRF/5 (nAUC = 0.446 ± 0.07), correlated with S2 or S3 Hoshida transcriptomic subclasses (Fig. 2C). A Tukey multiple comparisons test examining the significance of the differences between the survival AUC of the liver cancer cell lines supported the observation that PLC/PRF/5 and HepG2 exhibited the sensitive phenotype compared with SNU-449, SNU-423, SNU-387, and SK-Hep1 (Supplementary Table S4).

To capture each cell line’s transcriptomic landscape at high resolution, baseline RNA-seq was performed. PCA of RNA expression profiles of all cell lines demonstrated clustering of the five most resistant cell lines by nAUC (SK-Hep1, SNU-449, SNU-475, SNU-387, and SNU-423) along the second and third principal components, which comprised 13.1% and 8.2% of total variance across cell lines, respectively (Fig. 2D). A clear separation of the five most ^90^Y-resistant (SK-Hep1, SNU-387, SNU-449, SNU-423, and SNU-475) from the three most sensitive cell lines (PLC/PRF/5, Hep3B, and HepG2) was demonstrated along the second component of variance. Of note, two cell lines failed to cluster in either group, SNU-398 (nAUC 0.685 ± 0.105) and MHCC-97H (nAUC 0.702 ± 0.084), and demonstrated intermediate sensitivity to ^90^Y treatment. These findings suggest that intrinsic transcriptomic diversity contributes to variable cytotoxic responses to ^90^Y *in vitro*.

### Baseline transcriptomic signatures associated with ^90^Y resistance

**Figure 3.**
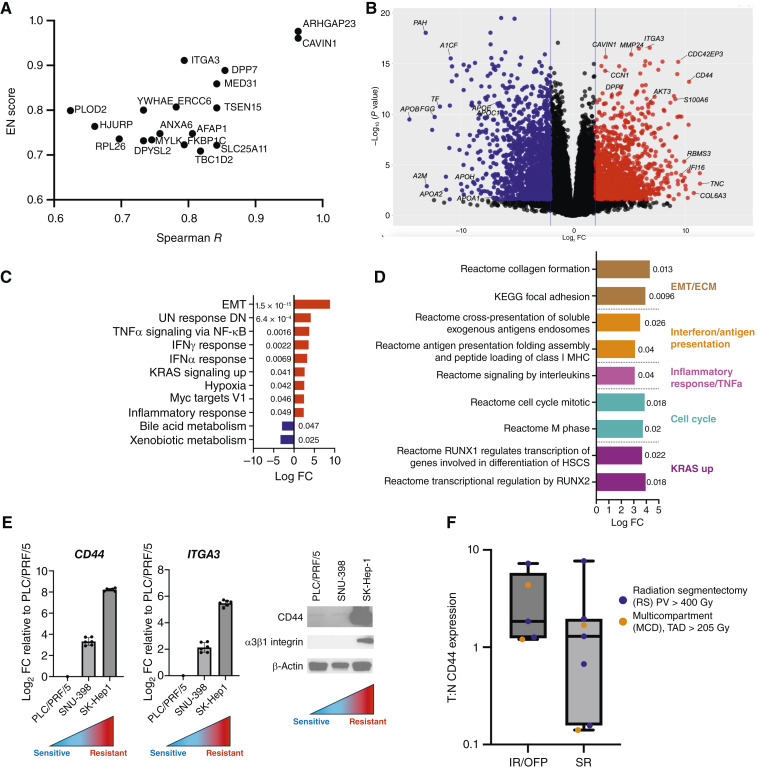
EMT and adhesion pathways associated with ^90^Y resistance. **A,** EN regression analysis identified 18 protein-encoding genes with nonzero coefficients for which expression was correlated with ^90^Y resistance across all cell lines at baseline (EN score >0.7). Among these, *ITGA3* (EN = 0.911, R = 0.79) encodes the α subunit of the α3β1 integrin heterodimer, previously reported to influence HCC tumor progression and immune checkpoint expression. **B,** Differential expression analysis of RNA expression between ^90^Y-resistant (SK-Hep1, SNU-449, SNU-475, SNU-387, and SNU-423) and -sensitive (PLC/PRF/5, Hep3B, and HepG2) cell lines. Groupings defined *a priori* by nAUC *Z*-scores and baseline PCA. Volcano plot of log_2_ FC vs. −log_10_*P* value (FDR adjusted) of genes upregulated (red) and downregulated (blue) in ^90^Y-resistant vs. -sensitive cell lines. Genes involved in the extracellular matrix (*ITGA3*) and cancer stemness (*CD44*) were significantly upregulated in ^90^Y-resistant cell lines. **C,** GSEA of Hallmark pathways demonstrates strong upregulation of the EMT pathway in ^90^Y-resistant cell lines (mean log FC 8.9), which contains *CD44* and *ITGB1*, the counterpart of *ITGA3* in the a3b1 integrin heterodimer. Numbers next to each gene set bar represent FDR. **D,** Consistent with Hallmark EMT enrichment, KEGG and Reactome pathways associated with extracellular matrix and integrin cell surface interactions are enriched in resistant cell lines. **E,** qPCR (mean log_2_ FC, error bars represent SEM, with *n* = 2 biological replicates) and Western blot validation of ITGA3/a3b1 and CD44 confirming elevated expression of these genes in the most ^90^Y-resistant (SK-Hep1) vs. ^90^Y-sensitive (PLC/PRF/5) and intermediate (SNU-398) cell lines, consistent with an EMT/adhesion phenotype associated with ^90^Y resistance. **F,** qPCR of tumor vs. normal CD44 expression demonstrates a trend toward higher CD44 expression in those with IR or OFP (*n* = 5) vs. SR (*n* = 12), although not powered for statistical significance (*P* = 0.43 Mann–Whitney). Color circle indicates treatment intent: blue, radiation segmentectomy; orange, multicompartment dosimetry (MCD) with TAD > 205 Gy. MIRD, medical internal radiation dose. Relative expression from qPCR data = 2^−(Cttarget − Cthousekeeping)^, in which Ct is the detection crossing threshold.

To link the expression of baseline genes with *in vitro*^90^Y-microsphere sensitivity, we applied EN regression with bootstrapping across all 10 cell lines. This analysis identified 18 protein-encoding genes for which expression correlated with ^90^Y resistance across all cell lines (EN score >0.7), including *ITGA3* (EN score = 0.911, R = 0.79), a subunit of the α3β1 integrin heterodimeric complex implicated in HCC progression and immune checkpoint expression (Fig. 3A). Additionally, differential expression of RNA-seq comparing the resistant and the sensitive groups revealed distinct genetic signatures corresponding to resistance and sensitivity, with upregulation of *ITGA3* and *CD44*, an extracellular matrix marker of cancer stemness (Fig. 3B; refs. 29–31). Hallmark GSEA revealed strong enrichment of the epithelial–mesenchymal transition (EMT) pathway in ^90^Y-resistant cell lines [pathway mean log fold change (FC) 8.9], which contains *CD44* and *ITGB1*, the counterpart of *ITGA3* in the α3β1integrin heterodimer, along with the enrichment of pathways related to the TNAα signaling, IFNγ, and IFNα and downregulation of bile acid/xenobiotic metabolism (Fig. 3C; ref. 32). Furthermore, cross-referencing with additional curated gene sets confirmed enrichment of Kyoto Encyclopedia of Genes and Genomes (KEGG) and Reactome pathways associated with extracellular matrix, integrin cell surface interactions, and antigen presentation in resistant cell lines (Fig. 3D). Protein and transcript validation confirmed upregulation of *ITGA3*/α3β1 and *CD44* in resistant (SK-Hep1) relative to intermediate (SNU-398) and sensitive (PLC/PRF/5) cell lines (Fig. 3E; Supplementary Fig. S1A). The most resistant lines also downregulated APO family genes with known tumor-suppressive roles (Fig. 3B; Supplementary Fig. S1B; refs. 33, 34). Overall, these data are consistent with an EMT phenotype with a baseline level of interferon signaling associated with resistance to ^90^Y microsphere treatment in our model.

To explore translational relevance, we tested whether tumor *CD44* expression would correlate with TARE clinical outcomes in a small exploratory cohort of patients for whom pretreatment tissue was available (Fig. 1A and B). Patients who experienced IR or OFP within 12 months (*n* = 5) trended toward higher *CD44* tumor:normal liver RNA expression levels compared with sustained responders (*n* = 7; median 1.841 vs. 1.295; *P* = 0.43 using Mann–Whitney), with all patients below background expression achieving SR (Fig. 3F). Although this difference was not statistically significant because of small sample size, the directionality aligns with our cell line data and suggests that CD44 expression may reflect or contribute to biological resistance. These results are hypothesis generating and warrant validation in larger clinical cohorts.

### Human liver cancer cell lines exhibit differential gene expression in response to ^90^Y treatment

**Figure 4.**
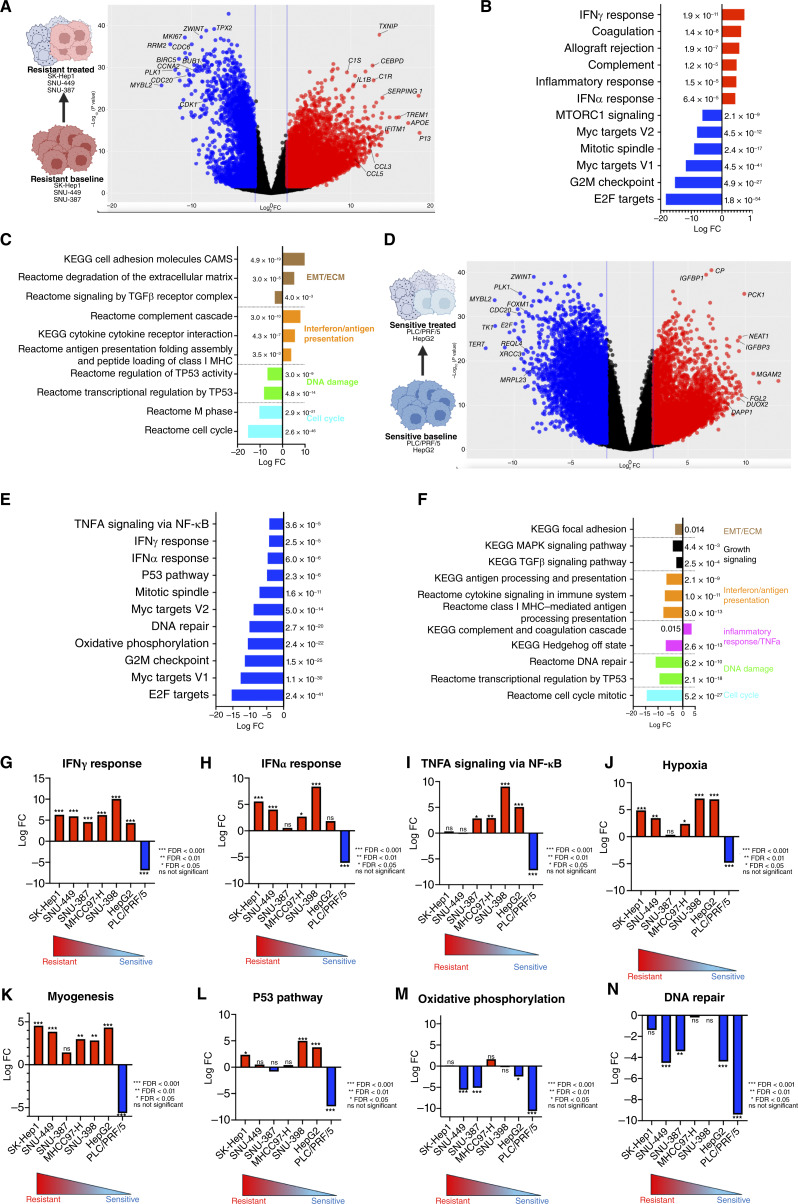
^90^Y-resistant and -sensitive cell lines demonstrate distinct patterns of biological process enrichment after ^90^Y microsphere treatment. **A,** Volcano plot of log_2_ FC vs. −log_10_*P* value of genes upregulated (red) and downregulated (blue) after ^90^Y microsphere treatment in select ^90^Y-resistant cell lines (SK-Hep1, SNU-449, and SNU-387). **B,** GSEA of Hallmark pathways demonstrates upregulation of pathways involved with inflammation and immune response after treatment in the ^90^Y-resistant group, such as IFNγ response (mean log_2_FC = 7.4), among others. **C,** Consistent with Hallmark IFNγ and IFNα enrichment, KEGG and Reactome pathways associated with cytokine signaling and antigen presentation were upregulated after treatment with ^90^Y. **D,** Volcano plot of log_2_ FC vs. −log_10_*P* value of genes upregulated (red) and downregulated (blue) after ^90^Y microsphere treatment in select ^90^Y-sensitive cell lines (PLC/PRF/5 and HepG2). **E,** GSEA of Hallmark pathways demonstrates downregulation of many of the inflammatory and immune response–related pathways that were upregulated in the resistant cell lines, including IFNγ response (mean log_2_ FC = −4.3,) and IFNα response (mean log_2_ FC = −4.7). No Hallmark pathways demonstrated significant upregulation after treatment in ^90^Y-sensitive cells. **F,** KEGG and Reactome pathway analysis corroborates downregulation of interferon- and antigen presentation–associated pathways, along with those involved with growth signaling such as MAPK (mean log FC = −4.01) and TGFβ (mean log FC = −2.62). Numbers next to each gene set bar represent FDR. Significance set at FDR <0.05 and log_2_ FC > 2. Heterogeneous activation of stress and survival pathways after ^90^Y microsphere treatment across different liver cancer cell lines. Significant variation in Hallmark gene sets (**G**) IFNγ, (**H**) IFNα, (**I**) TNFα signaling, (**J**) hypoxia, (**K**) myogenesis, (**L**) p53 pathway, (**M**) oxidative phosphorylation, and (**N**) DNA repair, showing heterogeneity in stress and survival pathway activation across cell lines after ^90^Y microsphere treatment.

To investigate treatment-induced adaptations, we performed RNA-seq in select cell lines after treatment with 20 MBq ^90^Y microspheres in independent experiments. Resistant lines (SK-Hep1, SNU-449, and SNU-387) showed marked upregulation (log_2_ FC > 10) of complement-modulating genes (*C1S*, *C1R*, and *SERPING1*) and stress/inflammatory mediators (*IL1B*, *IFITM1*, *CCL3*, and *CCL5*; Fig. 4A; ref. 35). Hallmark GSEA analysis of treated resistant cell lines confirmed upregulation of IFNγ and IFNα responses and complement and inflammatory responses pathways (mean log FC > 5) with concurrent downregulation of mTORC1, oxidative phosphorylation, Myc, and E2F pathways (Fig. 4B and C; Supplementary Fig. S2A). More granular analysis of KEGG and Reactome gene sets also showed upregulation of complement, cytokine signaling, and antigen presentation gene sets after treatment (Fig. 4C). *TXNIP*, which typically functions as a tumor suppressor, was also upregulated in ^90^Y-resistant cells, possibly reflecting an adaptive response to oxidative stress or altered glucose metabolism, given concurrent downregulation of DNA synthesis and cell-cycle progression genes (*BIRC5*, *RRM2*, *MKI67*, and *CDC6*). Taken together, these findings suggest metabolic adaptation as a potential component of the survival phenotype observed in ^90^Y-resistant cell lines.

In contrast, sensitive cell lines (PLC/PRF/5 and HepG2) demonstrated upregulation of genes related to reactive oxygen species generation and DNA damage, such as *CP*, *MGAM2*, *DUOX2*, *IGFBP3*, and *PCK1*, but lacked enrichment of adaptive stress pathways (Fig. 4D). Many of the inflammatory and immune response–related pathways that were upregulated in the resistant cell lines were downregulated after treatment in the sensitive cell lines, such as IFNγ and IFNα responses, along with those involved with growth signaling, including MAPK (mean log FC = −4.01) and TGFβ (mean log FC = −2.62; Fig. 4E and F; Supplementary Fig. S2B). When resistant and sensitive cell lines were both compared after treatment, several important patterns emerged. As expected, enrichment of genes associated with baseline signatures of resistance, such as EMT, IFNγ response, and IFNα response, was observed (Supplementary Fig. S3A and S3B). However, resistant lines maintained or induced survival after treatment, such as DNA repair, cell cycle checkpoints, and oxidative phosphorylation, whereas these were downregulated in sensitive cell lines (Supplementary Fig. S3C). Overall, ^90^Y-resistant cells seem capable of metabolic and inflammatory adaption to β-emitter–induced oxidative stress, whereas sensitive cells exhibit oxidative injury without compensatory survival responses.

Finally, Hallmark pathway analysis after ^90^Y microsphere treatment revealed wide heterogeneity across cell lines, with the most significant variation noted in the hypoxia, TNFα, IL6/JAK/STAT3, IFNγ response, p53 pathway, EMT, and DNA repair pathways, among others (Fig. 4G–N; Supplementary Fig. S4A–S4N). Notably, the intermediate-sensitivity line SNU-398 displayed robust activation of IFNγ response (mean log FC = 10), IFNα response (mean log FC = 8.4), and TNFα signaling (mean log FC = 9) pathways, distinguishing it from both resistant or sensitive groups. (Supplementary Fig. S4G and S4H). Overall, these data underscore extensive biological diversity in stress and damage response to ^90^Y among liver cancer cell lines.

### qRT-PCR validation differentially expressed genes after ^90^Y microsphere treatment

**Figure 5.**
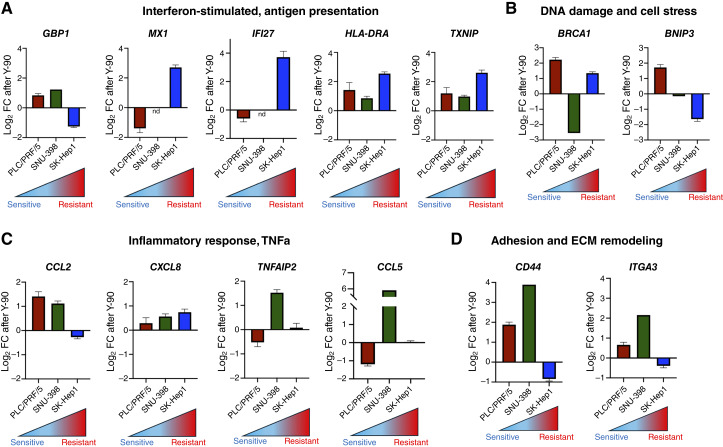
qRT-PCR validation of gene expression representing relevant biological pathways after ^90^Y microsphere treatment in the most sensitive (PLC/PRF/5), resistant (SK-Hep1), and intermediate (SNU-398) cell lines. Select genes involved in (**A**) interferon stimulation and antigen presentation were mostly upregulated in both SNU-398 and SK-Hep1 after treatment, with the exception of *GBP1* in SK-Hep1 and MX1 and IFI27 in SNU-398 which did not have reliable qPCR readouts. *TXNIP* was strongly upregulated in SK-Hep1 after treatment, consistent with increased oxidative stress signaling. (**B**) SNU-398 demonstrated very strong upregulation of inflammatory response genes, in particular *CCL5* and *TNFAIP2*. **C,** Distinct patterns of DNA damage and cell stress genes were seen across cell lines, with SK-Hep1 showing upregulation of *BRCA1* and downregulation of *BNIP3*. **D,** ECM genes *CD44* and *ITGA3* were strongly upregulated in SNU-398 after treatment, suggesting stress-induced EMT acquisition. Although there was slight downregulation of these genes in SK-Hep1, they remained at highest abundance in this line consistent with an EMT-associated expression profile. All experiments were performed in technical triplicate and error bars represent SEM when >1 biological replicate was performed. nd, no reliable qRT-PCR readout.

To validate transcriptomic findings, we assessed representative genes from resistance-associated pathways after treatment in select cell lines using qRT-PCR. These included interferon-/stress-related genes (*MX1*, *IFI27*, *HLA-DRA*, and *TXNIP*), inflammatory mediators (*GBP1*, *CCL5*, *CCL2*, and *TNFAIP2*), DNA damage/stress regulators (*BRCA1* and *BNIP3*), and EMT/adhesion markers (*CD44* and *ITGA3*). In the resistant SK-Hep1 line, interferon-related genes (*MX1*, *IFI27*, and *HLA-DRA*) and *TXNIP* were markedly upregulated after ^90^Y microsphere treatment, whereas they were downregulated or modestly increased in the sensitive PLC/PRF/5 cell line (Fig. 5A). Of note, *GBP1* was downregulated in SK-Hep1 after treatment, indicating selective rather than uniform activation of interferon-stimulated genes in this context. Strong upregulation of genes involved with TNFα signaling and inflammatory response was seen after treatment in the intermediate SNU-398 line, including *CCL5* (up to 6 log FC) and *TNFAIP2*, in line with GSEA results (Fig. 5B). Meanwhile, these genes were either unchanged or downregulated in the resistant Sk-Hep1 and PLC/PRF/5 lines, respectively. After treatment, distinct patterns of DNA damage and cell stress gene activation were seen (Fig. 5C). The sensitive PLC/PRF/5 cell line upregulated both *BRCA1* and *BNIP3*, suggesting induction of both DNA repair and *BNIP3*-related apoptotic pathways, consistent with decreased viability. Meanwhile, resistant cells upregulate *BRCA1* but suppress *BNIP3*, suggesting repair without mitochondrial apoptosis. Finally, both *CD44* and *ITGA3* were upregulated after treatment in the SNU-398 line, suggesting a stress-induced EMT acquisition (Fig. 5D). Although there was modest downregulation of both genes in the resistant SK-Hep1 line after treatment, their absolute abundance remained the highest of the tested cell lines, indicating a persistent EMT state (Supplementary Fig. S5).

## Discussion

HCC remains a leading cause of cancer-related death worldwide, underscoring the need for therapies that are both effective and biologically informed. TARE has been incorporated as an effective treatment within several practice guidelines worldwide across HCC stages (3, 7–9, 36). Despite this, response rates can vary despite well-accepted dosimetry benchmarks with persistent hazard of early OFP, both of which may be attributed to HCC’s molecular heterogeneity (5, 10, 13). Molecularly informed indicators of treatment resistance could improve patient selection and inform strategies to enhance lethality, which are especially relevant in the early and intermediate stages in which adjuvant combinatorial approaches with systemic therapies have been proposed (37, 38). Despite their growth and efficacy, investigations of molecular indicators of resistance to β-emitting therapeutic radioisotopes such as ^90^Y are notably sparse. This may be in part due to the logistic and handling challenges of working with these isotopes in laboratory settings.

In this discovery-oriented study, we derived a high-throughput pipeline for assessing viability and downstream biological pathway changes after escalating activities of commercially used glass ^90^Y microspheres adapted from others in a panel of human liver cell lines (25–27). Here, we reveal marked heterogeneity of survival across these 10 cell lines, with ^90^Y resistance corresponding to Hoshida S1 and C1 subtypes, along with enrichment of EMT and adhesion-related gene sets. This heterogeneity was supported by clear separation of nAUC values (an indicator of resistance to ^90^Y in our model) across cell lines and transcriptomic clustering of resistant versus sensitive lines on PCA. Key findings from the bulk RNA-seq were corroborated by increased mRNA and protein expression of *CD44* and *ITGA3*/α3β1 integrin within cell lines of increasing ^90^Y resistance. CD44 is a hyaluronan receptor and marker of cancer stemness involved with tumor initiation and termination of the p53 genomic surveillance response, making it a plausible mediator of radiation resistance (39). The integrin α3β1 binds to the extracellular matrix protein laminin, facilitating HCC cell adhesion and motility. This interaction supports EMT and enhances the metastatic potential of the tumor by engaging the intracellular signaling cascade downstream from focal adhesion kinase and PI3K/Akt (40, 41). For both CD44 and α3β1, the antibodies used detect the biologically active surface-expressed forms, including relevant isoforms and heterodimeric complexes, linking these proteins directly to EMT and adhesion pathways. Together, this positions the EMT/adhesion axis as a candidate molecular indicator of treatment resistance and a pathway of interest for future therapeutic exploration or noninvasive monitoring given its cell surface predominance (42, 43). Furthermore, integrating molecular insights with advanced dosimetry may in the future help refine understanding of dose–response relationships and biologically resistant compartments. TARE exists alongside other liver-directed radiotherapies such as stereotactic body radiotherapy and high–dose rate brachytherapy (44). Placing our findings within this broader radiotherapy landscape highlights opportunities to integrate molecular signatures with dose rate, perfusion-based predictors of response and rational combination strategies to improve clinical outcomes (45).

Another key finding was that, among the various liver cancer cell lines, treatment-induced pathways were also variable following ^90^Y treatment. Resistant cell lines mounted interferon, inflammatory, and antigen presentation programs with concomitant downregulation of cell cycle pathways, whereas sensitive lines downregulated interferon, DNA repair, and oxidative phosphorylation pathways. These findings were validated with qRT-PCR in independent experiments, confirming divergent treatment-induced pathways among sensitive and resistant lines. Notably, SNU-398, which did not cluster to either resistant or sensitive groups, exhibited strong induction of an inflamed and adhesive stress response noted by induction of *CCL5*, *GBP1*, and *TNFAIP2*, among others, and was distinct from the interferon-stimulated but chemokine-limited pattern seen in the resistant SK-Hep1 line. This reflects the intrinsic molecular diversity of HCC not only in viability after ^90^Y treatment but in downstream activation of stress, immune, and adhesion pathways, underscoring that response to ^90^Y is biologically nonuniform. These observations may have implications for future studies evaluating ^90^Y in combination with immune checkpoint inhibitors as *CCL5* has been implicated as playing a key role in antitumor immune responses after TARE (46).

In addition to the strengths and novelty of this work, we recognize several limitations. First, our studies were conducted primarily *in vitro* and thus cannot recapitulate a complex tumor microenvironment or the likely dose heterogeneity encountered *in vivo*. Viability was measured by MTS assay as a short-term surrogate, and although this approach has been previously validated for high-throughput assessment of radiation-based treatments, clonogenic survival may better capture therapeutic response to treatment (25). However, clonogenic assays were not feasible because they require very low–density plating, which is incompatible with the heterogeneous distribution of Y-90 microspheres and β-particle range, and would introduce substantial variability and radiologic handling challenges across multiple activities and cell lines. Accordingly, we placed emphasis on pathway patterns rather than formal radiosensitivity metrics. A further limitation is the cell line panel utilized, which largely represents the proliferative HCC subclass (frequently *TP53* mutant), given the lack of availability of cell lines representing the nonproliferative subtype (21). As such, generalizability of treatment resistance and transcriptional changes across cell lines should not be extrapolated to clinical dosimetry or outcomes. Rather, we suggest that our findings serve to generate hypotheses about biomarkers and pathways relevant to minimal residual/resistant disease (12, 47). More extensive protein-level quantification of CD44 and ITGA3/α3β1, including replicated Western blot or surface expression assays, will be an important next step for future mechanistic studies. Transcriptomic profiling and viability were performed at a single 10-day time point and fixed microsphere-specific activity, so transcriptional patterns unique to earlier or later time points and dose rate effects may also have been missed. Although underpowered, the trends observed in our studies of human liver samples are directionally concordant with our preclinical data linking EMT/adhesion programs (CD44/α3β1) to relative resistance. These observations suggest that *CD44* may be a candidate molecular indicator of ^90^Y response that warrants validation in larger, harmonized cohorts.

In summary, our high-throughput platform demonstrates that liver cancer cell lines display marked heterogeneity of response to ^90^Y microspheres. Our findings highlight EMT/adhesion and interferon-linked modules as candidate pathways associated with resistance, supporting future validation in functional and translational studies. Importantly, integrating such molecular insights with advanced dosimetry may ultimately help refine dose–response models and guide rational design of combination strategies to optimize patient outcomes.

## Supplementary Material

Supplemental Methods Detailed methods on cell culture, cell viability assays, RNAseq, western blot, and Elastic net regression analysis.

Supplemental Table S1 Details of liver cancer cell lines

Supplemental Table S2 Primer sequences for genes used for RT-qPCR

Supplemental Table S3 Demographics and clinical characteristics of exploratory patient cohort.

Supplemental Table S4 ANOVA results comparing nAUC between cell lines.

Supplemental Figure S1 Relative expression of CD44, ITGA3, and Apo genes.

Supplemental Figure S2 Full results of GSEA of Hallmark pathway analysis after 90Y microsphere treatment in resistant and sensitive cell lines.

Supplemental Figure S3 Differential gene expression between 90Y-resistant and sensitive cell lines after 90Y microsphere treatment

Supplemental Figure S4 Differential expression and GSEA after 90Y treatment in individual cell lines.

Supplemental Figure S5 Overall abundance of key genes before and after 90Y treatment.

## Data Availability

RNA-seq data generated for this study have been deposited in the Gene Expression Omnibus in compliance with MIAME/MINSEQE guidelines under the accession number GSE312144. Processed data tables, normalized expression matrices, and analysis scripts used in this study are available from the corresponding author upon reasonable request. All RNA-seq analyses were performed by the Genome Technology Access Center at Washington University using standard, open-source Bioconductor packages (including DESeq2 and limma/voom), as detailed in the Methods. Because the analyses used established open-source tools and no custom code was developed, the study does not generate stand-alone custom scripts. All other materials, including ^90^Y microsphere experimental protocols and cell line metadata, are described in the article and Supplementary Materials.
