# Supplementary material for: Epithelial–Mesenchymal Transition and Stress Adaptations Underlie Yttrium-90 Resistance in Liver Cancer Cell Lines
Source: Cancer Res Commun. 2026 Jan 22;6(1):178–90. doi: 10.1158/2767-9764.CRC-25-0627 (PMC12824473; doi:10.1158/2767-9764.CRC-25-0627)
Supplement: Supplemental Methods — Detailed methods on cell culture, cell viability assays, RNAseq, western blot, and Elastic net regression analysis. [file crc-25-0627_supplemental_methods_suppsm.docx]

**Supplemental Methods**

**Cell Line Culture**

SK-Hep-1 was routinely cultured in Dulbecco's Modified Eagle Medium (DMEM, Thermo Fisher Scientific, Waltham, MA) supplemented with 10% heat-inactivated fetal bovine serum (FBS, Thermo Fisher Scientific, Waltham, MA) and 1% Penicillin/Streptomycin (Pen/Strep, GIBCO, Thermo Fisher Scientific, Waltham, MA). Hep-3B2, Hep-G2/C3A, and PLC/PRF/5 were routinely cultured in Eagle's Minimal Essential Medium (EMEM, Sigma-Aldrich, St. Louis, MO) supplemented with 10% FBS and 1% Pen/Strep. MHCC-97H, SNU-387, SNU-398, SNU-423, SNU-449, and SNU-475 were routinely cultured in Roswell Park Memorial Institute 1640 medium (RPMI-1640, ThermoFisher Scientific, Waltham, MA) supplemented with 10% FBS and 1% Pen/Strep. All cell lines were maintained in a jacketed, humidified incubator with 5% CO2 at 37 °C and passaged when they became confluent.

**Cell Viability Assay**

Cell lines were plated at a density of 4x10^5^ per well in 24-well plates (TPP, Midwest Scientific, St. Louis, MO) in 1 mL of cell line-specific media and placed in the 37°C incubator with a humidified 5% CO_2_ atmosphere. After 16 hours, the cell culture inserts (Millicell Standing Cell Culture “standing” inserts for 24-well plate, PCF 0.4 µm pore, 1-2 mm leg height, Sigma-Aldrich, St. Louis, MO) were placed in each well. Each insert was filled with 200 µL of cell-matched media. ^90^Y-microspheres were added to the inserts at activity concentrations of 0 – 20 MBq/mL, and the plates were placed in the 37°C incubator, maintaining 5% CO2 and a humidified atmosphere for 10 days. Each ^90^Y-microsphere activity concentration was performed in triplicate. At the end of the incubation period, the inserts containing the ^90^Y-microspheres were discarded. The remaining cells were assessed for viability with a colorimetric assay. After treatment, the media was removed from each well and replaced with 0.5 mL of PBS containing 100 µL tetrazolium compound (3-(4,5-dimethylthiazol-2-yl)-5-(3-carbooxymethoxyphenyl)-2-(4-sulfophenyl)-2H-tetrazolium, inner salt; MTS) and phenazine ethosulfate (PES, electron coupling reagent) (CellTiter 96® Aqueous One Solution Cell Proliferation Reagent, Promega, Madison, WI) to determine the percentage of live cells. The plates were incubated at 37°C for 1-4 hours to allow the tetrazolium compound to develop. The resulting absorbance was detected at 490 nm with a Synergy/NEO2 multi-mode reader (BioTek, RRID:SCR_019765, Winooski, VT). Each cell line was assessed with at least three independent experiments, and the cell viability was expressed as % of untreated control cells. The exception was SNU-475, which was lost after one viability and baseline RNA-seq experiment but was included in our baseline experimental analysis.

**RNA extraction, sequencing, and analysis from liver cancer cell lines**

In collaboration with the Genome Technology Access Center (GTAC) at Washington University School of Medicine (RRID:SCR_001030), RNA samples were enriched using either PolyA selection or ribosomal depletion, yielding adequate RNA (>500 ng) or Takara Clontech SMARTer cDNA generation (<500 ng). RNA samples with an RNA integrity number (RIN) above 8.0 were sequenced. Illumina sequencing adapters were applied to the resulting cDNA ends, followed by amplification and sequencing on an Illumina NovaSeq 6000 using paired reads that extended 150 bases. RNA-seq reads were aligned and quantified using the Ensembl release 101 primary assembly with an Illumina DRAGEN Bio-IT server running version 3.9.3-8 software. All gene counts were imported into the R/Bioconductor package EdgeR (RRID:SCR_012802), and TMM normalization size factors were calculated to adjust for sample differences in library size.^1^ Ribosomal genes and genes not expressed in the smallest group size minus one samples, with greater than one count-per-million, were excluded from further analysis. The TMM size factors and the matrix of counts were imported into the R/Bioconductor package Limma ((RRID:SCR_010943).^2^ Weighted likelihoods based on the observed mean-variance relationship of every gene and sample were calculated for all samples and the count matrix and transformed to moderated log 2 counts-per-million with Limma’s voomWithQualityWeights.^3^ The performance of all genes was assessed with plots of the residual standard deviation of every gene to their average log-count with a robustly fitted trend line of the residuals. Differential expression analysis was performed to identify differences between conditions, and the results were filtered to include only those genes with Benjamini-Hochberg false-discovery rate-adjusted p-values of 0.05 or less. Alternatively, total RNA integrity was determined using an Agilent Bioanalyzer or a 4200 Tapestation. Library preparation was performed with 10ng of total RNA with a Bioanalyzer RIN score greater than 8.0. ds-cDNA was prepared using the SMARTer Ultra Low RNA kit for Illumina Sequencing (Takara-Clontech) per the manufacturer's protocol. cDNA was fragmented using a Covaris E220 sonicator with peak incident power of 18, duty factor of 20%, and 50 cycles per burst for 120 seconds. cDNA was blunt-ended, had an A base added to the 3' ends, and then had Illumina sequencing adapters ligated to the ends. Ligated fragments were then amplified for 12-15 cycles using primers incorporating unique dual index tags. Fragments were sequenced on an Illumina NovaSeq X Plus using paired-end reads extending 150 bases. Basecalls and demultiplexing were performed with Illumina’s bcl2fastq software (RRID:SCR_015058) and a custom Python demultiplexing program with a maximum of one mismatch in the indexing read. RNA-seq reads were then aligned to the Ensembl release 101 primary assembly with STAR version 2.7.9a (RRID:SCR_004463).^4^ Gene counts were derived from the number of uniquely aligned, unambiguous reads by Subread:featureCount version 2.0.3.^5^ Isoform expression of known Ensembl transcripts was quantified with Salmon version 1.5.2.^6^ Sequencing performance was assessed for the total number of aligned reads, the total number of uniquely aligned reads, and the features detected. The ribosomal fraction, known junction saturation, and read distribution over known gene models were quantified with RSeQC version 4.0 (RRID:SCR_005275).^7^ For each contrast extracted with Limma, global perturbations in known Gene Ontology (GO) terms, MSigDb, and KEGG pathways were detected using the R/Bioconductor package GAGE^8^ to test for changes in expression of the reported log 2 fold-changes reported by Limma in each term versus the background log 2 fold-changes of all genes found outside the respective term. The R/Bioconductor package heatmap3^9^ was used to display heatmaps across groups of samples for each GO or MSigDb term with a Benjamini-Hochberg false-discovery rate-adjusted p-value less than or equal to 0.05. Perturbed KEGG pathways (RRID:SCR_012773) where the observed log 2 fold-changes of genes within the term were significantly perturbed in a single direction versus background or in any direction compared to other genes within a given term, with p-values less than or equal to 0.05, were rendered as annotated KEGG graphs with the R/Bioconductor package Pathview (RRID:SCR_002732).^10^ To find the most critical genes, the Limma voomWithQualityWeights transformed log 2 counts-per-million expression data was then analyzed via weighted gene correlation network analysis with the R/Bioconductor package WGCNA.^11^ Briefly, all genes were correlated with each other by Pearson correlations and clustered by expression similarity into unsigned modules using a power threshold empirically determined from the data. An eigengene was then created for each de novo cluster, and its expression profile was correlated across all coefficients of the model matrix. Because these clusters of genes were produced by expression profile rather than known functional similarity, the clustered modules were given the names of random colors, where grey is the only module that has any pre-existing definition of containing genes that do not cluster well with others. These de novo clustered genes were then tested for functional enrichment of known GO terms with hypergeometric tests available in the R/Bioconductor package clusterProfiler (RRID:SCR_016884).^12^ Significant terms with Benjamini-Hochberg adjusted p-values less than 0.05 were then collapsed by similarity into clusterProfiler category network plots to display the most significant terms for each module of hub genes, facilitating the interpretation of each significant module's function. The information for all clustered genes for each module was then combined with their respective statistical significance results from Limma to determine whether or not those features were also found to be significantly differentially expressed.

For RT-qPCR experiments, total RNA was isolated for each treated cell line using NucleoSpin RNA plus with DNA removal column (Macherey-Nagel, Duren, Germany). Total RNA was then transcribed to cDNA with SuperScript III First-strand cDNA system (Invitrogen) to verify expression of genes and verify knockdown efficiencies using Fast Sybr Green Master Mix (Invitrogen) as per the manufacturer’s protocol.

**RNA isolation from human liver tissues**

Tissue samples were homogenized using a Bullet Blender and 1 mm zirconium oxide beads (Next Advance, Troy, NY) in TRIzol reagent (Invitrogen, Grand Island, NY). Once homogenization was completed, RNA was processed using the manufacturer’s guidelines. DNase-treated RNAs (RNAse-free DNase kit, Qiagen, Germantown, MD) underwent cDNA synthesis using random hexamers and MultiScribe Reverse Transcriptase from High-Capacity cDNA Reverse Transcription kit (Applied Biosystems, Foster City, CA, USA). Quantitative evaluation of RNA abundance was performed using Fast SYBR Green Master Mix (Applied Biosystems, Foster City, CA) in a Step One Plus Real Time PCR system instrument (Applied Biosystems, Foster City, CA). RT-qPCR was performed in triplicate for each sample. Human RNA levels were normalized to human 18S in each sample and expressed as fold changes.

**Western Immunoblot Assay**

Untreated cells were mechanically dissociated from 24-well plates using rubber-tipped cell scrapers (Sarstedt, Newton, NC) and washed 3 times with PBS. Cells were then incubated with 25 mM Tris-HCl pH 7.6, 150 mM NaCl, 1% nonyl phenoxypolyethoxylethanol (NP-40), 0.5% sodium deoxycholate, 0.1% sodium dodecyl sulfate (SDS) buffer (RIPA lysis buffer, Abcam, RRID:SCR_012931, Cambridge, MA) supplemented with EDTA-free protease inhibitor cocktail (Pierce, Thermo Fisher, Waltham, MA) for 60 minutes at 4°C followed by centrifugation at 12,000 x g for 10 minutes. Protein content was quantified using the BCA protein assay (Pierce, Thermo Fisher Scientific, Waltham, MA). Lysates (50 µg total protein) were denatured for 5 minutes at 100 °C, and proteins were separated by sodium dodecyl sulfate polyacrylamide gel electrophoresis (SDS-PAGE) at 100 V for 1.5 hours using Mini-PROTEAN TG precast gels with Tris/Glycine/SDS buffer system (Bio-Rad, RRID:SCR_008426, Hercules, CA). The proteins were transferred to a polyvinylidene fluoride membrane at 50V for 2 hours (Immobilon-P, Millipore, RRID:SCR_008983, Burlington, MA). After blocking for 1 hour in TBST blocking buffer (10 mM Tris-Cl, 150 mM NaCl, and 0.05% [v/v] Tween-20, pH 7.5, 5% (w/v) non-fat milk), the polyvinylidene difluoride (PVDF) membrane was rinsed once with TBST and incubated overnight at 4 °C with 1° antibody diluted in TBST blocking buffer. After 2 washes, 5 minutes each, the membrane was incubated with 2° antibodies conjugated with horseradish peroxidase (HRP) for 1 hour at room temperature. The unbound secondary antibody was removed with three washes in TBST, 10 minutes each. Bound 2° antibodies were detected using SuperSignal^TM^ West Pico Plus Detection Substrate (ThermoFischer Scientific, Waltham, MA) and ChemiDoc^TM^ Imager (BioRad, Hercules, CA). The following primary antibodies were used for immunoblotting: CD44 (Rat / IgG2b monoclonal, kappa, 1 µg/mL, unconjugated, clone IM7, #14-0441-82, RRID:AB_467246, ThermoFisher, Waltham, MA); CD49c/CD29 (Rabbit IgG polyclonal, 1:300, unconjugated, #BS-1057R, RRID:AB_3718176, Thermo Fisher, Walthon, MA); ß-actin (1:1000, unconjugated, clone C4, sc-47778, RRID:AB_626632, Bio-Rad, Hercules, CA). The following secondary antibodies were used for immunoblotting: goat anti-rabbit IgG-HRP (1:5000, #31430, RRID:AB_228307, ThermoFisher Scientific, Waltham, MA), goat anti-rabbit IgG-HRP (1:5000, #31460, RRID:AB_228341, ThermoFisher Scientific, Waltham, MA), goat anti-rat IgG-HRP (1:5000, #31470, RRID:AB_228356, ThermoFisher Scientific, Waltham, MA).

**Elastic Net Regression Analysis**

The raw dataset contained 60,871 genes. Rows with zero counts across all columns were filtered out, leaving 49,681 genes. The averages of triplicate gene counts were calculated for each gene across different cell lines, followed by calculating the mean of the normalized Area Under the Curve values (nAUC) representing the viability profiles for each cell line. The averaged gene counts and nAUC means were paired to obtain Spearman correlation coefficients (R) between the averaged gene counts and the nAUC means. Genes with a Spearman R greater than 0.2 were retained, resulting in 12,447 genes. Fragments Per Kilobase Million (FPKM) values were then read and processed, and only genes with FPKM values greater than 0.1 for all 30 samples were retained, leaving 3,152 genes. To implement the EN regression analysis, a prediction matrix with a dimension of 10×3,152 was standardized to have a zero mean and unit standard deviation for each feature. The response variables were the nAUC means. These were fitted for the EN regression using the R packages glmnet version 4.1.8 and “caret” version 6.0.94. A 10-fold cross-validation with five repetitions was used to optimize the α and λ parameters, aiming to minimize the root mean squared error (RMSE). This process involved 20 potential α values, ranging from 0.01 to 0.2, and 250 potential λ values, spanning the range from e^-5^ to e^5^. The best α was found to be 0.02, and the best λ was 0.085. After parameter optimization, a bootstrapping procedure was employed with the optimal α and λ to generate 1,000 resampled datasets by sampling with replacement. For each bootstrap sample, the EN regression model was fitted, and the regression coefficients were extracted. A list of regression coefficients (β) was obtained and used to build the predictive score.

**Supplemental References**

1. Robinson MD, McCarthy DJ, Smyth GK. edgeR: a Bioconductor package for differential expression analysis of digital gene expression data. *Bioinformatics* 2010; **26**(1): 139-40.

2. Ritchie ME, Phipson B, Wu D, et al. limma powers differential expression analyses for RNA-sequencing and microarray studies. *Nucleic Acids Res* 2015; **43**(7): e47.

3. Liu R, Holik AZ, Su S, et al. Why weight? Modelling sample and observational level variability improves power in RNA-seq analyses. *Nucleic Acids Res* 2015; **43**(15): e97.

4. Dobin A, Davis CA, Schlesinger F, et al. STAR: ultrafast universal RNA-seq aligner. *Bioinformatics* 2013; **29**(1): 15-21.

5. Liao Y, Smyth GK, Shi W. featureCounts: an efficient general purpose program for assigning sequence reads to genomic features. *Bioinformatics* 2014; **30**(7): 923-30.

6. Patro R, Duggal G, Love MI, Irizarry RA, Kingsford C. Salmon provides fast and bias-aware quantification of transcript expression. *Nat Methods* 2017; **14**(4): 417-9.

7. Wang L, Wang S, Li W. RSeQC: quality control of RNA-seq experiments. *Bioinformatics* 2012; **28**(16): 2184-5.

8. Luo W, Friedman MS, Shedden K, Hankenson KD, Woolf PJ. GAGE: generally applicable gene set enrichment for pathway analysis. *BMC Bioinformatics* 2009; **10**: 161.

9. Zhao S, Guo Y, Sheng Q, Shyr Y. Advanced heat map and clustering analysis using heatmap3. *Biomed Res Int* 2014; **2014**: 986048.

10. Luo W, Brouwer C. Pathview: an R/Bioconductor package for pathway-based data integration and visualization. *Bioinformatics* 2013; **29**(14): 1830-1.

11. Langfelder P, Horvath S. WGCNA: an R package for weighted correlation network analysis. *BMC Bioinformatics* 2008; **9**: 559.

12. Yu G, Wang LG, Han Y, He QY. clusterProfiler: an R package for comparing biological themes among gene clusters. *OMICS* 2012; **16**(5): 284-7.
