# Supplementary material for: Epithelial–Mesenchymal Transition and Stress Adaptations Underlie Yttrium-90 Resistance in Liver Cancer Cell Lines
Source: Cancer Res Commun. 2026 Jan 22;6(1):178–90. doi: 10.1158/2767-9764.CRC-25-0627 (PMC12824473; doi:10.1158/2767-9764.CRC-25-0627)
Supplement: Supplemental Table S1 — Details of liver cancer cell lines [file crc-25-0627_supplemental_table_s1_suppst1.docx]

**Supplemental Table S1**. Key details of liver cancer cell lines used in this study.

| **Cell Line** | **Cellosaurus ID** | **Liver Disease Etiology** | **Ethnicity** | **Gender** | **Relevant Mutated Genes** |
| --- | --- | --- | --- | --- | --- |
| **HepG2/C3A** | **CVCL_1098** | **None** | **European** | **Male** | **TERT, CTNNB1** |
| **Hep3B** | **CVCL_0326** | **HBV** | **African American** | **Male** | **TP53** |
| **PLC/PRF/5** | **CVCL_0485** | **HBV** | **African** | **Male** | **TP53, CDKN2A** |
| **MHCC-97H** | **CVCL_4972** | **HBV** | **Asian** | **Male** | **TP53, CTNNB1** |
| **SNU-387** | **CVCL_0250** | **HBV** | **Asian** | **Female** | **TP53, TERT, CDKN2A** |
| **SNU-398** | **CVCL_0077** | **HBV** | **Asian** | **Male** | **TP53, TERT, CTNNB1** |
| **SNU-423** | **CVCL_0366** | **HBV** | **Asian** | **Male** | **TP53, TERT** |
| **SNU-449** | **CVCL_0454** | **HBV** | **Asian** | **Male** | **TP53, CDKN2A** |
| **SNU-475** | **CVCL_0497** | **HBV** | **Asian** | **Male** | **TP53, TERT** |
| **SK-Hep-1** | **CVCL_0525** | **None** | **European** | **Male** | **BRAF, CDKN2A** |
