# Supplementary material for: Epithelial–Mesenchymal Transition and Stress Adaptations Underlie Yttrium-90 Resistance in Liver Cancer Cell Lines
Source: Cancer Res Commun. 2026 Jan 22;6(1):178–90. doi: 10.1158/2767-9764.CRC-25-0627 (PMC12824473; doi:10.1158/2767-9764.CRC-25-0627)
Supplement: Supplemental Table S2 — Primer sequences for genes used for RT-qPCR [file crc-25-0627_supplemental_table_s2_suppst2.docx]

**Supplemental Table S2.** Primer sequences for genes used for RT-qPCR

|  |  |
| --- | --- |
| **Gene name** | **Sequence** |
| CD44 F | CTCCAGTGAAAGGAGCAGCA |
| CD44 R | AGCAGGGATTCTGTCTGTGC |
| ITGA3 F | GGGTACACGATGCAGGTAGG |
| ITGA3 R | CTCCTGGCTCAGCAAGAACA |
| GBP1 F | ACTTCAGGAACAGGAGCAACT |
| GBP1 R | ACATGCCTTTCGTCGTCTCA |
| BRCA1 F | GCTTTTCAGCTTGACACAGGT |
| BRCA1 R | TCCTGAGTTTTCATGGACAGCA |
| HLA-DRA F | GGAGTCGGTCCTGGGGAATA |
| HLA-DRA R | AAGCCCAAATGAACCGGGAT |
| STING1 F | GACAGAGCCTACCAGTTGGG |
| STING1 R | CATTTGTGTGCTGGCTCAGG |
| MX1 F | TGCGCAGGGACCGGAAT |
| MX1 R | CGGCGTTCTTCACTCCAGAT |
| IFI27 F | GGACTCTGGAATGTCTGGCT |
| IFI27 R | TGGTCACTGCTGATGAGGTG |
| IL1B F | GCCCAGCCAAGACGGTTATT |
| IL1B R | AGCCCTCCCTTTTGTATTGAGG |
| CXCL8 F | ACCCCAAGGAAAACTGGGTG |
| CXCL8 R | GGCTGACTTTGGATTATATGTCTCA |
| CCL2 F | CCATGGACCACCTGGACAAG |
| CCL2 R | AGGGTGTCTGGGGAAAGCTA |
| CCL5 F | CTGCTTTGCCTACATTGCCC |
| CCL5 R | TCGGGTGACAAAGACGACTG |
| TXNIP F | CAACTTGCTGCCCGACAAAA |
| TXNIP R | TGGGTGGCATGCAAGGTATT |
| TNFAIP2 F | CTTTTCCTGTGGCCTGACCT |
| TNFAIP2 R | GAACCAGCGCTGCTCTTAAC |
| CCN5 F | GTGCCTCTGTAAGCAGGACC |
| CCN5 R | GTGCTCCATTCTGGGCAGG |
| BNIP3 F | CAACCTCCACCAGCACCTTT |
| BNIP3 R | GGCCACCCCAGGATCTAACA |
| Patient tissue CD44 Fwd | GATCATCTTGGCATCCCTCTTG |
| Patient tissue CD44 Rev | CGACTGTTGACTGCAATGCA |
| Human 18S Fwd | CGAACGTCTGCCCTATCAACTT |
| Human 18S Rev | ACCCGTGGTCACCATGGTA |
