# Supplementary material for: Epithelial–Mesenchymal Transition and Stress Adaptations Underlie Yttrium-90 Resistance in Liver Cancer Cell Lines
Source: Cancer Res Commun. 2026 Jan 22;6(1):178–90. doi: 10.1158/2767-9764.CRC-25-0627 (PMC12824473; doi:10.1158/2767-9764.CRC-25-0627)
Supplement: Supplemental Table S3 — Demographics and clinical characteristics of exploratory patient cohort. [file crc-25-0627_supplemental_table_s3_suppst3.docx]

**Supplemental Table S3.** Demographics and clinical characteristics of exploratory patient cohort.

HCV – hepatitis C virus, MASLD – Metabolic Dysfunction-Associated Steatotic Liver Disease, FFPE - formalin-fixed paraffin-embedded, RS – radiation segmentectomy, MCD – multicompartment dosimetry, TAD – tumor absorbed dose, IR – infield recurrence, OFP – out of field progression, CR – complete response, PR – partial response
