# Supplementary material for: Epithelial–Mesenchymal Transition and Stress Adaptations Underlie Yttrium-90 Resistance in Liver Cancer Cell Lines
Source: Cancer Res Commun. 2026 Jan 22;6(1):178–90. doi: 10.1158/2767-9764.CRC-25-0627 (PMC12824473; doi:10.1158/2767-9764.CRC-25-0627)
Supplement: Supplemental Table S4 — ANOVA results comparing nAUC between cell lines. [file crc-25-0627_supplemental_table_s4_suppst4.docx]

**Supplemental Table S4.** ANOVA results comparing nAUC between cell lines.

| **Tukey's multiple comparisons test** | **Summary** | **Adjusted P value** |
| --- | --- | --- |
| HepG2   vs. SNU-449 | ns | 0.1804 |
| HepG2   vs. PLC/PRF/5 | ns | 0.2137 |
| HepG2   vs. SNU-423 | ns | 0.7955 |
| HepG2   vs. SNU-387 | ns | 0.497 |
| HepG2   vs. SK-Hep 1 | * | 0.0212 |
| HepG2   vs. SNU-398 | ns | >0.9999 |
| HepG2   vs. MHCC97-H | ns | 0.9997 |
| HepG2   vs. Hep3B | ns | >0.9999 |
| HepG2   vs. SNU-475 | ns | 0.7926 |
| SNU-449   vs. PLC/PRF/5 | *** | 0.0007 |
| SNU-449   vs. SNU-423 | ns | 0.9919 |
| SNU-449   vs. SNU-387 | ns | >0.9999 |
| SNU-449   vs. SK-Hep 1 | ns | 0.9948 |
| SNU-449   vs. SNU-398 | ns | 0.4577 |
| SNU-449   vs. MHCC97-H | ns | 0.5943 |
| SNU-449   vs. Hep3B | ns | 0.342 |
| SNU-449   vs. SNU-475 | ns | >0.9999 |
| PLC/PRF/5   vs. SNU-423 | * | 0.0124 |
| PLC/PRF/5   vs. SNU-387 | ** | 0.0043 |
| PLC/PRF/5   vs. SK-Hep 1 | **** | <0.0001 |
| PLC/PRF/5   vs. SNU-398 | ns | 0.1531 |
| PLC/PRF/5   vs. MHCC97-H | ns | 0.1029 |
| PLC/PRF/5   vs. Hep3B | ns | 0.217 |
| PLC/PRF/5   vs. SNU-475 | ns | 0.0561 |
| SNU-423   vs. SNU-387 | ns | >0.9999 |
| SNU-423   vs. SK-Hep 1 | ns | 0.7116 |
| SNU-423   vs. SNU-398 | ns | 0.9638 |
| SNU-423   vs. MHCC97-H | ns | 0.9895 |
| SNU-423   vs. Hep3B | ns | 0.914 |
| SNU-423   vs. SNU-475 | ns | >0.9999 |
| SNU-387   vs. SK-Hep 1 | ns | 0.9419 |
| SNU-387   vs. SNU-398 | ns | 0.7984 |
| SNU-387   vs. MHCC97-H | ns | 0.8924 |
| SNU-387   vs. Hep3B | ns | 0.6871 |
| SNU-387   vs. SNU-475 | ns | >0.9999 |
| SK-Hep 1 vs. SNU-398 | ns | 0.0977 |
| SK-Hep 1 vs. MHCC97-H | ns | 0.1524 |
| SK-Hep 1 vs. Hep3B | ns | 0.0627 |
| SK-Hep 1 vs. SNU-475 | ns | 0.9994 |
| SNU-398 vs. MHCC97-H | ns | >0.9999 |
| SNU-398 vs. Hep3B | ns | >0.9999 |
| SNU-398 vs. SNU-475 | ns | 0.9234 |
| MHCC97-H vs. Hep3B | ns | >0.9999 |
| MHCC97-H vs. SNU-475 | ns | 0.9602 |
| Hep3B vs. SNU-475 | ns | 0.8736 |
