# Supplementary material for: Epithelial–Mesenchymal Transition and Stress Adaptations Underlie Yttrium-90 Resistance in Liver Cancer Cell Lines
Source: Cancer Res Commun. 2026 Jan 22;6(1):178–90. doi: 10.1158/2767-9764.CRC-25-0627 (PMC12824473; doi:10.1158/2767-9764.CRC-25-0627)
Supplement: Supplemental Figure S1 — Relative expression of CD44, ITGA3, and Apo genes. [file crc-25-0627_supplemental_figure_s1_suppsf1.docx]

**Supplemental Figure S1**

**Supplemental Figure S1**. **A)** Relative expression of CD44 and ITGA3 to housekeeping gene by RT-qPCR, (each point is technical replicate amongst 2 biological replicates). **B)** RT-qPCR (mean log2 fold change) of Apo family genes confirming decreased expression in intermediate (SNU-398) and resistant (SK-Hep1) compared to sensitive (PLC/PRF/5) cells. nd=no reliable RT-qPCR readout
