# Supplementary material for: Epithelial–Mesenchymal Transition and Stress Adaptations Underlie Yttrium-90 Resistance in Liver Cancer Cell Lines
Source: Cancer Res Commun. 2026 Jan 22;6(1):178–90. doi: 10.1158/2767-9764.CRC-25-0627 (PMC12824473; doi:10.1158/2767-9764.CRC-25-0627)
Supplement: Supplemental Figure S2 — Full results of GSEA of Hallmark pathway analysis after 90Y microsphere treatment in resistant and sensitive cell lines. [file crc-25-0627_supplemental_figure_s2_suppsf2.docx]

**Supplemental Figure S2**

**Supplemental Figure S2**. Full results of GSEA of Hallmark pathway analysis after ^90^Y microsphere treatment in **A)** resistant (SK-Hep1, SNU-387, SNU-449) and **B)** sensitive (PLC/PRF/5, HepG2) cell lines. Numbers next to each gene set bar represent FDR. Significance set at FDR< 0.05 and log2FC > 2.0.
