# Supplementary material for: Epithelial–Mesenchymal Transition and Stress Adaptations Underlie Yttrium-90 Resistance in Liver Cancer Cell Lines
Source: Cancer Res Commun. 2026 Jan 22;6(1):178–90. doi: 10.1158/2767-9764.CRC-25-0627 (PMC12824473; doi:10.1158/2767-9764.CRC-25-0627)
Supplement: Supplemental Figure S3 — Differential gene expression between 90Y-resistant and sensitive cell lines after 90Y microsphere treatment [file crc-25-0627_supplemental_figure_s3_suppsf3.docx]

**Supplemental Figure S3**

**Supplemental Figure S3**. Differential gene expression between ^90^Y-resistant and sensitive cell lines after ^90^Y microsphere treatment illustrate divergent adaptive pathways. **A)** Volcano plot of log_2_FC versus -log10 p value of gene expression changes (red-upregulated, blue-downregulated in ^90^Y-resistant vs sensitive cell lines). **B)** Gene set enrichment analysis of Hallmark pathways demonstrates enrichment of processes characterizing ^90^Y-resistant cell lines at baseline, such as EMT (mean log_2_FC 10.8), Interferon Gamma Response (mean log_2_FC 10.1), and Interferon Alpha Response (mean log_2_FC 8.4). In addition, processes involved in pro-survival responses after radiation, such as DNA Repair (mean logFC 6.3) and cell cycle checkpoints were significantly upregulated in ^90^Y-resistant cell lines after treatment. **C)** KEGG and Reactome pathway analysis further corroborates this, with ^90^Y-resistant cell lines upregulating numerous pathways involved in antigen presentation, Interferon signaling, DNA damage, and oxidative phosphorylation.
