# Supplementary material for: Epithelial–Mesenchymal Transition and Stress Adaptations Underlie Yttrium-90 Resistance in Liver Cancer Cell Lines
Source: Cancer Res Commun. 2026 Jan 22;6(1):178–90. doi: 10.1158/2767-9764.CRC-25-0627 (PMC12824473; doi:10.1158/2767-9764.CRC-25-0627)
Supplement: Supplemental Figure S4 — Differential expression and GSEA after 90Y treatment in individual cell lines. [file crc-25-0627_supplemental_figure_s4_suppsf4.docx]

**Supplemental Figure S4**

**Supplemental Figure S4**. Gene expression changes (Volcano plots represent log2 fold change (log_2_FC) versus -log_10_ p value of genes upregulated (red) and downregulated (blue)) and Hallmark GSEA after ^90^Y microsphere treatment by individual cell line. **A,B)** PLC/PRF/5, **C,D)** HepG2, **E,F)** MHCC97-H, **G,H)** SNU-398, **I,J)** SNU-387, **K,L)** SNU-449, and **M,N)** SK-Hep1. PLC/PRF/5, the most sensitive cell line demonstrated downregulation of all listed pathways after treatment, in particular DNA repair and oxidative phosphorylation. SNU-398 demonstrated intermediate sensitivity to ^90^Y microsphere treatment and was not grouped in either resistant or sensitive groups based on nAUC Z-score or PCA clustering. This line demonstrated particularly strong upregulation of genes such as *TNFAIP2*, *IL6*, and *GBP1* and pathways involved with extracellular matrix organization, antigen presentation, TNFalpha signaling and inflammation. Significance set at FDR< 0.05 and log_2_FC > 2.0.
