# Supplementary material for: Epithelial–Mesenchymal Transition and Stress Adaptations Underlie Yttrium-90 Resistance in Liver Cancer Cell Lines
Source: Cancer Res Commun. 2026 Jan 22;6(1):178–90. doi: 10.1158/2767-9764.CRC-25-0627 (PMC12824473; doi:10.1158/2767-9764.CRC-25-0627)
Supplement: Supplemental Figure S5 — Overall abundance of key genes before and after 90Y treatment. [file crc-25-0627_supplemental_figure_s5_suppsf5.docx]

**Supplemental Figure S5**

**Supplemental Figure S5**. Overall abundance of genes representing key pathways indicated by relative expression to housekeeping gene across cell lines. Each point is a technical replicate among 1-2 biological replicates.
